# Snake Venom Peptide Fractions from *Bothrops jararaca* and *Daboia siamensis* Exhibit Differential Neuroprotective Effects in Oxidative Stress-Induced Zebrafish Models

**DOI:** 10.3390/ph18050678

**Published:** 2025-05-02

**Authors:** Felipe Assumpção da Cunha e Silva, Brenda Rufino da Silva, Leticia Ribeiro de Barros, Emidio Beraldo-Neto, Adolfo Luis Almeida Maleski, Carlos Alberto-Silva

**Affiliations:** 1 Experimental Morphophysiology Laboratory, Natural and Humanities Sciences Center (CCNH), Universidade Federal do ABC (UFABC), São Bernardo do Campo 09606-070, SP, Brazil; fhellcunha@gmail.com (F.A.d.C.e.S.); brenda.rufino@ufabc.edu.br (B.R.d.S.); ribeiro.leticia@aluno.ufabc.edu.br (L.R.d.B.); adolfomaleski@gmail.com (A.L.A.M.); 2 Biochemistry Laboratory, Butantan Institute, São Paulo 05503-900, SP, Brazil; emidio.beraldo@butantan.gov.br

**Keywords:** neuroprotection, PC12 cells, venom-derived peptides hydrogen peroxide, locomotor activity

## Abstract

**Introduction:** Snake venoms are rich sources of bioactive peptides with therapeutic potential, particularly against neurodegenerative diseases linked to oxidative stress. While the peptide fraction (<10 kDa) from *Bothrops jararaca* venom has shown in vitro neuroprotection, analogous fractions from related species remain unexplored in vivo. **Methods:** This study comparatively evaluated the neuroprotective effects of two peptide fractions (pf) from *Daboia siamensis* (pf-*Ds*) and *B. jararaca* (pf-*Bj*) against H_2_O_2_-induced oxidative stress using in vitro (PC12 cells) and in vivo (zebrafish, *Danio rerio*) models. **Results:** In vitro, pf-*Ds* (1 µg mL^−1^) did not protect PC12 cells against H_2_O_2_-induced cytotoxicity, unlike previously reported effects of pf-*Bj*. In vivo, neither pf-*Ds* nor pf-*Bj* (1–20 µg mL^−1^) induced significant developmental toxicity in zebrafish larvae up to 120 h post-fertilization (hpf). The neuroprotective effects of both pf were evaluated using two experimental models: (I) Larvae at 96 hpf were exposed to either pf-*Ds* or pf-*Bj* (10 µg mL^−1^) for 4 h, followed by co-exposure to H_2_O_2_ (0.2 mmol L^−1^) for an additional 10 h to induce oxidative stress (4–20 h model); (II) Embryos at 4 hpf were treated with pf-*D*s or pf-*Bj* (10 µg mL^−1^) continuously until 96 hpf, after which they were exposed to H_2_O_2_ (0.2 mmol L^−1^) for another 24 h (96–120 h model). In a short-term treatment model, neither fraction reversed H_2_O_2_-induced deficits in metabolism or locomotor activity. However, in a prolonged treatment model, pf-*Bj* significantly reversed the H_2_O_2_-induced locomotor impairment, whereas pf-*Ds* did not confer protection. **Conclusions:** These findings demonstrate, for the first time, the in vivo neuroprotective potential of pf-*Bj* against oxidative stress-induced behavioral deficits in zebrafish, contingent on the treatment regimen. The differential effects between pf-*Ds* and pf-*Bj* highlight species-specific venom composition and underscore the value of zebrafish for evaluating venom-derived peptides.

## 1. Introduction

Neurodegenerative diseases represent a substantial and increasing worldwide public health challenge, marked by the gradual degeneration of neurons and resulting functional decline [1]. Oxidative stress is a well-established etiological factor in the pathophysiology of these diseases [1,2], arising from a marked imbalance between excessive production of reactive oxygen species (ROS) and the diminished efficiency of endogenous antioxidant defense mechanisms [3]. Oxidative damage affects essential macromolecules, including lipids, proteins, and DNA, hence undermining neuronal function and survival [2]. This comprehension promotes the continuing search for innovative bioactive compounds that may provide neuroprotection by reducing oxidative damage and reestablishing cellular homeostasis. In this context, chemicals originating from animal venoms are attracting significant interest as abundant sources of peptides and proteins with different and potent pharmacological properties [4,5]. Snake venoms, specifically, represent intricate mixtures of bioactive components [6]. Beyond their roles in predation and defense, specific venom-derived peptides have demonstrated remarkable potential in modulating key cellular processes relevant to neurodegeneration [6,7]. These peptides can exhibit antioxidant, anti-inflammatory, and direct neuroprotective actions, making them attractive candidates for therapeutic development [5,7,8].

Components isolated from various snake venoms have been shown to influence neuronal survival pathways, attenuate apoptosis, and counteract the detrimental effects of ROS [7]. Research within the Viperidae family has provided compelling evidence for this potential. Notably, a low-molecular-mass peptide fraction (<10 kDa) isolated from the venom of *Bothrops jararaca* (pf-*Bj*) demonstrated significant protective effects against hydrogen peroxide (H_2_O_2_)-induced cytotoxicity in neuronal cell models, such as cultured hippocampal cells and neuronal PC12 neuronal-like cells [9,10]. Studies involving structurally related proline-rich oligopeptides (PROs) initially described from pf-*Bj* suggest the modulation of redox-sensitive signaling pathways and metabolic routes, potentially including the L-arginine pathway, contributes to these protective effects [8,9]. However, despite the wealth of data on *Bj*-derived PROs [9,10], there is a notable gap in the literature concerning the neuroprotective potential of analogous peptide fractions from the venom of other related vipers, such as *Daboia siamensis*. Given potential variations in venom composition and activity even among related species, exploring the peptide fraction from *D. siamensis* (pf-*Ds*) is warranted. Although in vitro data have established pf-*Bj* as a promising neuroprotective prototype [9,10], the specific composition of pf-*Ds* has not yet been fully characterized, highlighting a critical knowledge gap that limits our understanding of the molecular basis underlying its potential neuroprotective effects. These outcomes also underscore the need for validation in more physiologically complex systems. While in vitro models are essential for initial screening and for unraveling molecular mechanisms, they fall short in replicating the intricate systemic interactions, metabolic dynamics, and biological barriers of a whole organism. To address these limitations, in vivo animal models remain crucial.

The zebrafish (*Danio rerio*) has emerged as a powerful and versatile vertebrate model in biomedical and neuropharmacological research [11,12]. Its advantages include significant genetic homology with mammals, rapid external embryonic development, optical transparency of embryos facilitating real-time in vivo imaging, and suitability for behavioral assays relevant to neurological function [2,11,13]. Zebrafish have proven particularly robust for investigating oxidative stress mechanisms [2] and evaluating the efficacy and potential toxicity of neuroactive and neuroprotective compounds [12,14]. Studies utilizing zebrafish larvae have successfully modeled neuronal damage and assessed consequent functional deficits [15], underscoring their utility in understanding neurodegenerative processes and screening therapeutic interventions.

Therefore, this study aims to provide the first comparative evaluation of the neuroprotective effects of pf-*Ds* and pf-*Bj* venoms. By employing a dual-model approach, integrating in vitro assessments using the PC12 cell line with in vivo evaluations in the zebrafish model subjected to H_2_O_2_-induced oxidative stress, we seek to determine whether pf-*Ds* possesses neuroprotective activity comparable or distinct to the established effects of pf-*Bj*. This investigation intends to elucidate the potential of these under-explored venom-derived fractions, contributing valuable insights that may pave the way for the development of innovative therapeutic strategies against oxidative stress-mediated neurodegenerative disorders.

## 2. Results

### 2.1. Toxicological and Neuroprotective Effects of pf-Ds in PC12 Cells

**Figure 1:**
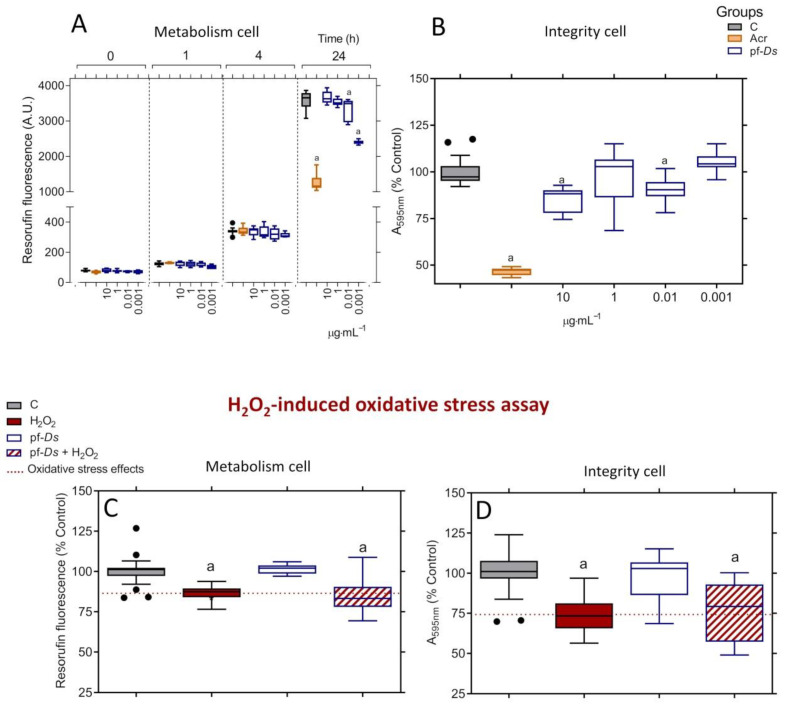
Toxicity profile of pf-*Ds* and its neuroprotective effects against oxidative stress-induced changes in neuronal PC12 cells. The viability of the cells was assessed by metabolism (**A**) and integrity (**B**) assays. The metabolic activity was measured after 0, 1, 4, and 24 h of treatment, and the integrity of the cell was analyzed after 24 h of treatment. The pf-*Ds* -mediated neuroprotection was evaluated by metabolism (**C**) and integrity (**D**) assays. The values were expressed as a percentage relative to the control, presented in box-and-whisker plots obtained from three independent sextuple experiments, and analyzed using one-way ANOVA, followed by a Tukey post-test. (a) Statistical significance was observed with *p* < 0.05 compared to the control group. C: untreated cell group; Acr: cell group treated with acrylamide.

Treatment with pf-*Ds* at concentrations of 0.01 and 0.001 μg mL^−1^ reduced the metabolic activity of PC12 cells, but only after 24 h of exposure. No significant effects were observed at concentrations equal to or higher than 1 μg mL^−1^ (Figure 1A). Additionally, pf-*Ds* impaired cell integrity at 0.01 and 10 μg mL^−1^, while no alterations were detected at 1 and 0.001 μg mL^−1^ (Figure 1B). Under the same treatment conditions, acrylamide (100 mmol L^−1^) markedly decreased both metabolism and cell integrity compared to the control (Figure 1A,B). Based on the absence of effects on metabolic activity and cell integrity, the concentration of 1 μg mL^−1^ pf-*Ds* was selected for subsequent neuroprotection assays. However, pf-*Ds* did not exhibit neuroprotective effects against H_2_O_2_-induced oxidative stress, as neither metabolic activity (Figure 1C) nor cell integrity (Figure 1D) showed improvement.

### 2.2. Toxicological Effects of pf-Ds and pf-Bj on Zebrafish Development

**Figure 2:**
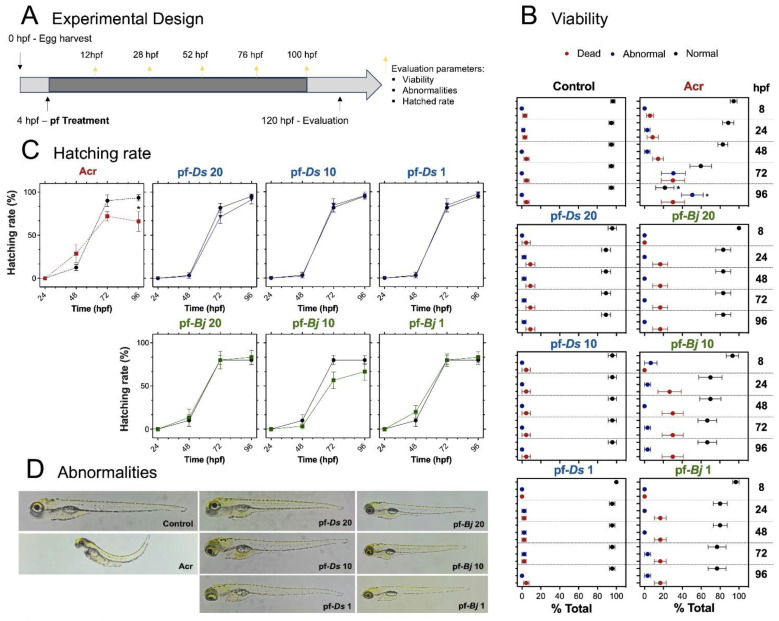
Toxicological effects of pf-*Ds* and pf-*Bj* on zebrafish development. (**A**) Experimental design: Embryos (4 hpf) were collected and exposed to different concentrations of pf-*Ds* or pf-*Bj* (20, 10, or 1 μg mL^−1^) in a 24-well plate (5 embryos per well in 500 μL of E3 solution). Acr (2 mmol L^−1^) was used as a positive was used as a positive control, and a separate untreated group served as the negative control. Assessments were conducted at 8, 24, 48, 72, and 96 hpf to evaluate abnormalities, hatching rate, and viability. (**B**) Viability assessment at 4 to 96 hpf showing the proportion of dead, abnormal, and normal larvae. (**C**) Hatching rate (%) over time in different treatment groups. (**D**) Representative images of abnormalities observed in different experimental groups compared to the control groups. One-way ANOVA and unpaired *t*-test were used for statistical evaluation (* *p* < 0.05). hpf: hours post-fertilization; Acr: Acrylamide.

The larvae were exposed to 20, 10, and 1 μg mL^−1^ concentrations of pf-*Ds* (represented by pf-*Ds* 20, pf-*Ds* 10, and pf-*Ds* 1, respectively) or pf-*Bj* (represented by pf-*Bj* 20, pf-*Bj* 10, and pf-*Bj* 1, respectively). Neither fraction exhibited toxic effects on zebrafish development. The treated groups did not show an increased incidence of mortality or morphological abnormalities. As expected, the group exposed to acrylamide showed significantly higher rates of lethality and malformations (Figure 2B). Similarly, pf-*D*s and pf-*Bj* had no effect on hatching rates when compared to the control group, whereas acrylamide treatment resulted in delayed hatching and a reduced overall hatching percentage (Figure 2C). Furthermore, neither pf-*Ds* nor pf-*Bj* induced visible malformations in zebrafish larvae, even at the highest concentrations tested. In contrast, the acrylamide-treated group presented clear developmental defects, including spinal curvature, yolk sac edema, and pericardial edema (Figure 2D).

However, the absence of gross morphological defects or lethality does not preclude specific modes of toxicity, such as neurotoxicity. Therefore, it was important to analyze key biochemical markers to ensure neurological safety. To this end, we assessed the activity of SOD, CAT, and AChE, critical enzymes in neurotransmission often targeted by neurotoxic compounds, in the exposed larvae [16].

**Figure 3:**
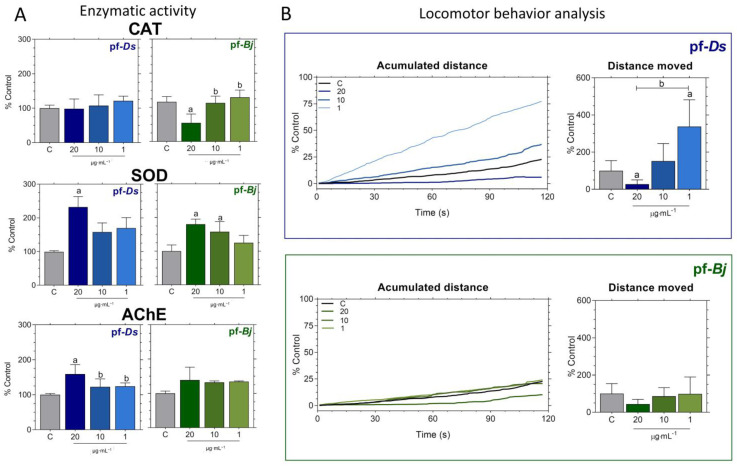
Effects of pf-*Ds* and pf-*Bj* on enzymatic activity and locomotor behavior in zebrafish embryos after 120 hpf. (**A**) Catalase (CAT), superoxide dismutase (SOD), and acetylcholinesterase (AChE) activities were measured and expressed as a percentage relative to the control group. (**B**) Behavioral analysis was obtained from accumulated distance and total distance moved in embryos exposed to the highest concentration of pf-*Ds* (20 μg mL^−1^), whereas pf-*Bj* treated groups showed no major alterations. Behavioral analyses were performed using ImageJ2 and Fiji. Statistical analyses were conducted using one-way ANOVA followed by Dunnett’s post-test and an unpaired *t*-test. Symbols indicate statistical significance: a (*p* < 0.05 vs. control) and b (*p* < 0.05 vs. other concentrations).

Overall, SOD activity exhibited treatment-dependent variations, with a significant increase at the highest pf-*Ds* concentration and at both pf-*Bj* 20 and pf-*Bj* 10 concentrations (Figure 3A). No relevant changes were observed in CAT expression (Figure 3A). The pf-*Ds* altered cholinergic function at its highest concentration (Figure 3A). No statistically significant differences in zebrafish larvae locomotor activity were observed compared to the control group; the group treated with pf-*Ds* 20 demonstrated a reduction in total distance traveled, suggesting possible neurotoxic or sedative effects, whereas pf-*Ds* 1 increased locomotor activity (Figure 3B). In contrast, pf-*Bj*-treated larvae did not significantly differ from controls, maintaining comparable swimming distances (Figure 3B).

### 2.3. Neuroprotective Effects of pf-Ds and pf-Bj Against H_2_O_2_-Induced Oxidative Stress in Zebrafish Embryos in 4–20 h Model

**Figure 4:**
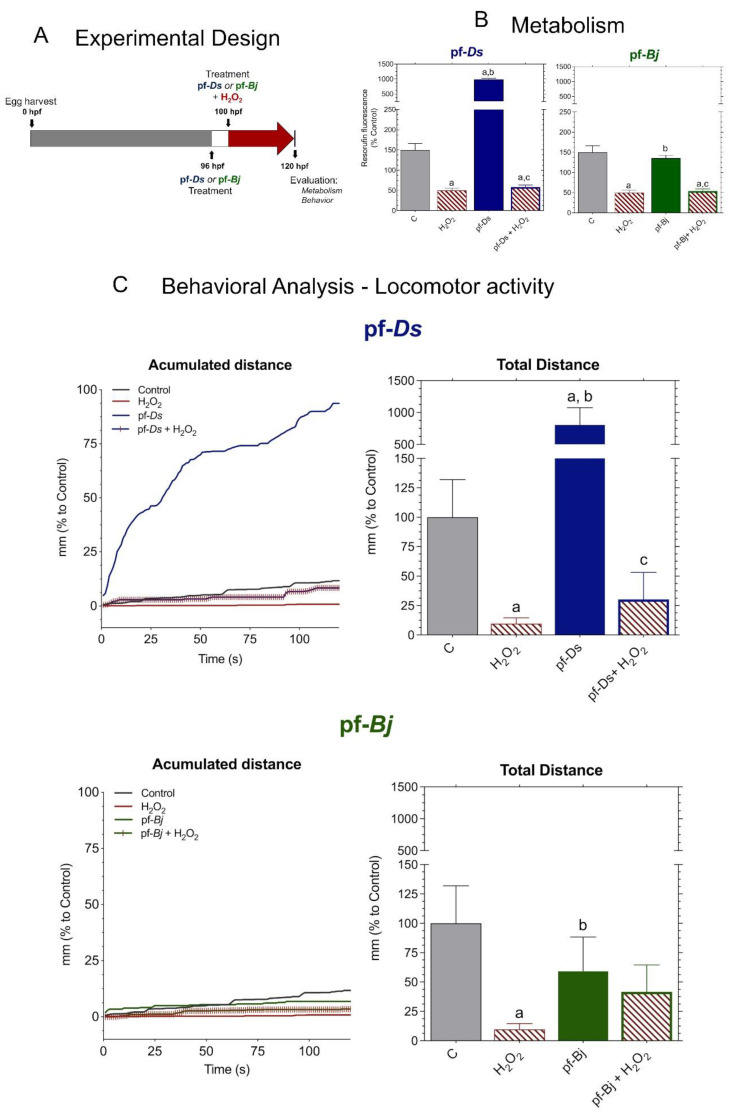
Neuroprotective effects of pf-*Ds* and pf-*Bj* against H_2_O_2_-induced oxidative stress in zebrafish in 4–20 h model. (**A**) Zebrafish embryos were harvested at 0 hpf and exposed to pf-*Ds* or pf-*Bj* (10 μg mL^−1^) at 96 hpf. At 100 hpf, embryos were co-treated with H_2_O_2_ (0.2 mmol L^−1^) to induce oxidative damage. At 120 hpf, metabolic and behavioral parameters were assessed to evaluate the neuroprotective potential of the fractions. (**B**) Metabolism was assessed through fluorescence emitted by resazurin reduction into resorufin, expressed as a percentage of the control group. Higher fluorescence intensity indicates increased cellular metabolism. (**C**) Locomotor activity was assessed based on the accumulated and total distance moved by embryos exposed to different treatments, using ImageJ2 and Fiji software. Statistical differences are indicated by: a (vs. Control), b (vs. H_2_O_2_), and c (vs. respective fraction alone) (*p* < 0.05), analyzed using one-way ANOVA followed by Dunnet’s post hoc test.

The pf-*Ds* 10 group presented the highest metabolic rate, significantly exceeding the control and other treatments. However, the presence of pf-*Ds* 10 was unable to reverse the oxidative stress caused by H_2_O_2_. The pf-*Bj* 10 group did not demonstrate significant metabolic activity relative to the control, nor was it able to counteract the effects of H_2_O_2_ in the pf-*Bj* 10 + H_2_O_2_ group (Figure 4B). The H_2_O_2_ group significantly reduced larval locomotion, likely due to decreased metabolism. Exposure to pf-*Ds* 10 resulted in a marked increase in total distance traveled, indicating neuromotor excitation, which aligns with the previously observed metabolic increase. By contrast, pf-*Bj* 10 showed only a slight reduction relative to controls, indicating a comparatively weaker effect on locomotion. Neither pf-*Ds* 10 nor pf-*Bj* 10 reversed the effects of the H_2_O_2_ group (Figure 4C).

### 2.4. Neuroprotective Effects of f-Ds and pf-Bj Against H_2_O_2_-Induced Oxidative Stress in Zebrafish Embryos in 96–120 h Model

**Figure 5:**
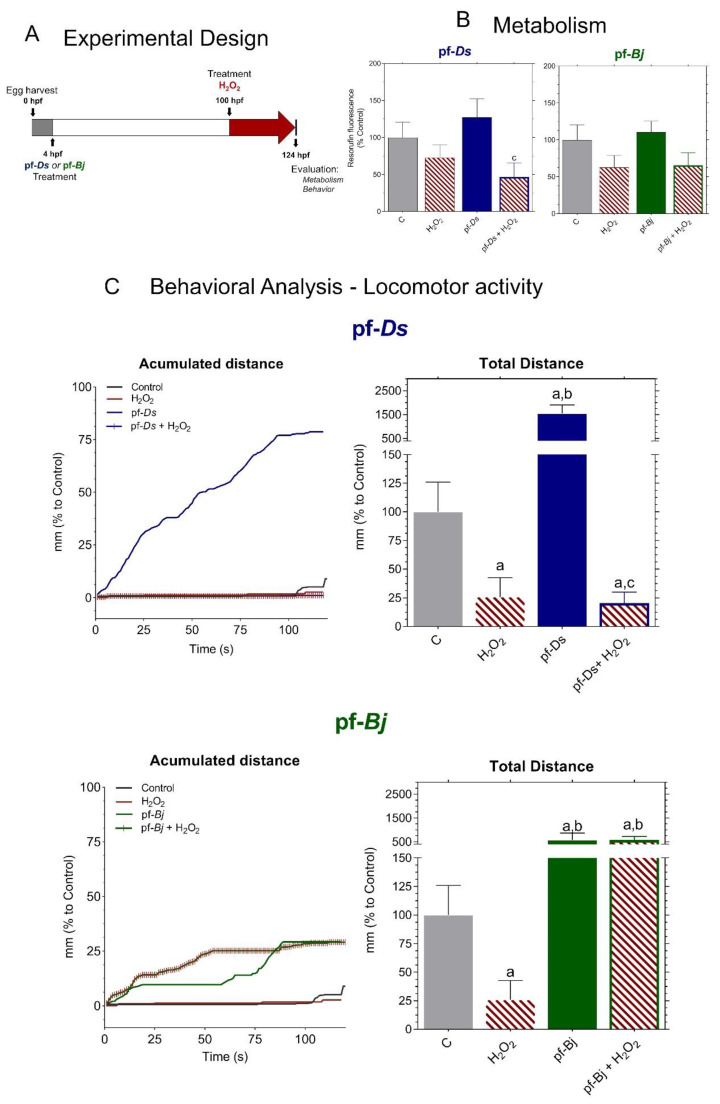
Neuroprotective effects of pf-*Ds* and pf-*Bj* against H_2_O_2_-induced oxidative stress in zebrafish embryos in 96–120 h model. (**A**) Zebrafish embryos were harvested at 0 hpf and exposed to pf-*Ds* or pf-*Bj* (10 μg mL^−1^) at 4 hpf. At 100 hpf, larvae were treated with H_2_O_2_ (0.2 mmol L^−1^) to induce oxidative damage. At 124 hpf, metabolic and behavioral parameters were assessed to evaluate the neuroprotective potential of the fractions. (**B**) Metabolic activity was assessed through fluorescence emitted by resazurin reduction into resorufin, expressed as a percentage of the control group. Higher fluorescence intensity indicates increased cellular metabolism. (**C**) Locomotor activity was assessed based on the accumulated and total distance moved by embryos exposed to different treatments, using ImageJ2 and Fiji software. Statistical differences (*p* < 0.05) are indicated by: a (vs. Control), b (vs. H_2_O_2_), and c (vs. respective fraction alone), analyzed using one-way ANOVA followed by Dunnett’s post hoc test.

We adapted the experimental protocol following established metabolic and behavioral assessment methods for zebrafish larvae (Figure 5A), according to methodologies described in the literature [17]. H_2_O_2_ treatment reduced metabolic activity, and, while pretreatment with pf-*Ds* 10 slightly increased metabolism compared to control levels, the fractions did not mitigate the metabolic impairment caused by H_2_O_2_ exposure (Figure 5B). Exposure to H_2_O_2_ led to a significant reduction in locomotion. Pre-treatment with pf-*Ds* 10 significantly increased locomotion compared to both the control and H_2_O_2_-treated groups but did not exhibit a protective effect against H_2_O_2_-induced damage. Notably, pre-treatment with pf-*Bj* 10, in addition to enhancing larval locomotion, was able to significantly reverse the lethargy induced by H_2_O_2_. The accumulated distance plots highlight the protective effects of pf-*Bj* 10 against H_2_O_2_-induced oxidative stress, as well as the increase in locomotor activity observed in larvae treated with pf-*Bj* 10 and pf-*Ds* 10. Treatment with pf-*Bj* 10 demonstrated a remarkable capacity to counteract the neurotoxic consequences induced by H_2_O_2_, thereby suggesting a potential neuroprotective mechanism (Figure 5C).

## 3. Discussion

The investigation of peptide fractions derived from snake venom has shown novel opportunities for the identification of neuroprotective compounds with potential pharmaceutical applications [9,18,19,20]; nonetheless, their in vivo neuroprotective effects remain mainly unexplored. This study presents the first evidence that the pf-*Bj* obtained from *B. jararaca* venom has neuroprotective efficacy against oxidative stress-induced toxicity in a neurodegenerative model using zebrafish, unlike pf-*Ds* derived from *D. siamensis* venom, given both being members of the Viperidae family.

Previous studies reported that the pf-*Bj* showed neuroprotective properties against H_2_O_2_-induced toxicity in primary cultured hippocampus cells [10], reducing SOD, caspase-3, and caspase-8 expressions. Additionally, pf-*Bj*-mediated neuroprotection was also demonstrated against oxidative stress in PC12 cells, but not in astrocyte-like C6 cells [9]. The neuroprotective mechanism of this fraction is proposed to depend on the L-arginine metabolism pathway, particularly through the production of polyamines (agmatine and spermidine), which are well documented for their role in neuroprotection [21,22,23,24,25,26]. Interestingly, our study demonstrates that pf-*Ds* did not restore H_2_O_2_-induced metabolic activity or cell integrity in PC12 cells, similar to the effects observed with pf-*Bj*. The geographical distribution of *D. siamensis* and *B. jararaca* may have an impact on the distinct compositions of the fractions, which could be responsible for this variation in neuroprotective effects on PC12 cells. Variations in venom composition due to environmental factors and evolutionary pressures across different regions may contribute to the divergent biological activities observed between these species, despite belonging to the same snake family, Viperidae [27].

The toxicological evaluation of novel bioactive compounds is a critical step in determining their potential for biomedical applications [28]. In this study, we assessed the toxicity of peptide fractions from *D. siamensis* and *B. jararaca* in *Danio rerio* larvae over a 96 h exposure period. Toxicity assessments in zebrafish models typically rely on morphological abnormalities, survival rates, and behavioral alterations [11]. Zebrafish larvae exposed to pf-*Ds* and pf-*Bj* did not exhibit significant morphological deformities, such as pericardial edema, spinal curvature, or yolk sac alterations, common indicators of developmental toxicity [14,29]. Furthermore, survival rates were comparable to those of the control group, suggesting that these peptide fractions do not induce acute lethality. The absence of toxic effects in our study is consistent with previous findings on bioactive peptides, where certain natural peptides have demonstrated safety in zebrafish models [29]. However, while no immediate toxicity was observed, further investigations are required to assess potential sublethal effects at the molecular and cellular levels, including oxidative stress markers and apoptotic pathways [30]. Overall, our results indicate that pf-*Ds* and pf-*Bj* exhibit a favorable safety profile in zebrafish larvae, reinforcing their potential for further exploration in neuroprotective and therapeutic applications. Future studies should focus on long-term exposure assessments and detailed biochemical analyses to fully elucidate their biological impact.

The assessment of enzymatic activities, particularly SOD, CAT, and AChE, is crucial for investigating neurodegeneration and neuroprotection [31]. SOD and CAT are key components of the endogenous antioxidant defense system, working together to neutralize ROS and mitigate oxidative stress, a major contributor to neurodegenerative processes [32]. A previous study identified that AChE in zebrafish is encoded by a single gene localized on linkage group 7, with its expression playing a crucial role in neuronal and muscular development during embryogenesis, which underscores the importance of AChE as a biomarker for neurodevelopmental studies [33,34]. In zebrafish, exposure to environmental toxins can significantly impact the activity of these enzymes [34,35]. Therefore, evaluating SOD, CAT, and AChE activities in zebrafish exposed to peptide fractions provides a comprehensive approach for assessing their potential neuroprotective properties, offering mechanistic insights into their roles in mitigating oxidative stress and preserving neural function. 

The enzymatic responses elicited by the peptide fractions highlight their favorable neurochemical safety profiles and underscore their promising potential for neuroprotective applications. The lack of alterations in CAT activity following pf-*Ds* exposure suggests that this fraction does not induce oxidative stress or redox imbalance [36], while the reduction in CAT observed only at the highest concentration of pf-*Bj* occurred without behavioral or metabolic disturbances, indicating no evidence of toxic oxidative burden [37]. Furthermore, the increase in SOD activity—observed at 20 µg·mL^−1^ for pf-*D*s and at 10 and 20 µg·mL^−1^ for pf-*Bj*—appears to reflect a controlled activation of antioxidant defenses, rather than a compensatory response to damage [38,39]. This pattern of enzymatic engagement, in the absence of adverse effects, reinforces the non-toxic nature of the fractions and suggests that they may contribute to cellular resilience under stress conditions [40]. Importantly, neither fraction caused AChE inhibition, a classic hallmark of neurotoxicity [41]. On the contrary, pf-*Ds* elicited an increase in AChE activity, which may indicate a modulatory, non-harmful influence on cholinergic signaling [42]. Together, these findings strongly support the conclusion that pf-*Ds* and pf-*Bj* are biochemically active yet non-toxic, establishing a solid foundation for their further exploration as candidate sources of neuroprotective compounds. Taken together, the enzymatic analyses suggest that both pf-*Ds* and pf-*Bj* do not induce neurotoxicity under the conditions tested. Instead, they exhibit distinct biochemical profiles that may reflect subtle neuromodulatory effects, particularly in redox signaling, without compromising enzymatic homeostasis. These findings position both peptide fractions as safe candidates for further bioprospecting efforts. This finding is consistent with previous reports indicating that natural peptides can exert bioactive properties without disrupting normal enzymatic functions [4,43]. However, the impact of prolonged exposure or repeated administration should be further investigated to rule out potential cumulative effects.

Locomotor behavior is an important factor in testing for neurotoxicity and neuroactivity because changes in movement can show how substances affect neurotransmission pathways [13]. Notably, although lower concentrations did not induce significant changes, exposure to the highest tested doses resulted in a marked increase in movement. In vitro assays alone cannot fully capture the effects of this behavioral alteration, which may reflect a modulation of neural circuitry and enzyme activity. Similar findings have been reported for bioactive peptides derived from marine organisms, which have demonstrated neuromodulatory effects in zebrafish models [44]. Taken together, these findings suggest that, while pf-*Ds* and pf-*Bj* do not induce overt toxicity, they may exert subtle neuromodulatory effects at higher concentrations. Furthermore, integrating behavioral data with morphological and molecular analyses enhances the utility of zebrafish as a neurotoxicology model, reinforcing their relevance for future studies on neuroactive compounds.

Building on the confirmed non-toxic profile of the tested fractions, we advanced to the zebrafish neuroprotection protocol to more comprehensively assess their efficacy in counteracting oxidative stress-induced metabolic impairment. In this in vivo model, exposure to hydrogen peroxide similarly results in a marked decline in metabolic activity, paralleling observations in cell-based systems. Interestingly, a clear divergence was observed between the two fractions: pf-*Ds* significantly enhanced metabolic activity, whereas pf-*Bj* did not produce any measurable effect. Although neither fraction fully reversed the peroxide-induced metabolic suppression, the robust stimulatory effect elicited by pf-*Ds* in zebrafish underscores the heightened sensitivity and translational relevance of the whole-organism model [37]. This heightened responsiveness is likely due to the intricate physiological architecture of zebrafish, which involves systemic interactions and compensatory mechanisms that are not present in isolated cell-based models [12].

The consistent metabolic decline induced by H_2_O_2_ in both PC12 cells and zebrafish supports the validity of the 4–20 h oxidative stress model across distinct biological systems. These findings further establish the zebrafish as a robust and translationally relevant model for investigating oxidative stress and assessing the efficacy of novel neuroprotective compounds, effectively bridging the gap between simplified in vitro assays and the complex responses of whole [12,45]. Exposure to pf-*Ds* and pf-*Bj* at 96 h post-fertilization (hpf) for a 24 h period failed to confer significant protection against oxidative stress, suggesting that short-term treatment at this developmental stage may be insufficient to trigger endogenous defense pathways or mitigate oxidative damage. This result is consistent with previous studies showing that brief interventions may not adequately activate neuroprotective mechanisms [46,47].

Given this lack of neuroprotection, we implemented an alternative approach in which the peptides were administered earlier, starting at 4 hpf, and maintained continuously until 100 hpf, followed by an additional 24 h exposure to H_2_O_2_. This prolonged exposure aimed to assess whether early and continuous treatment could enhance resilience against oxidative stress by promoting long-term cellular adaptations. Previous research has demonstrated that sustained exposure to neuroprotective agents during critical developmental windows can lead to improved neuronal outcomes [48,49]. The observed metabolic resilience suggests that, by 100 hpf, zebrafish larvae may have developed more mature and effective endogenous antioxidant systems, potentially mitigating the metabolic impact of oxidative stressors like H_2_O_2_. This observation aligns with studies indicating the progressive maturation of antioxidant defenses, such as the Nrf2/Keap1 (nuclear factor erythroid 2-related factor 2/Kelch-like ECH-associated protein 1) pathway, during zebrafish development [1,50,51,52]. The inability of pf-*Ds* and pf-*Bj* to significantly influence metabolism may also stem from their limited bioavailability or from specific molecular interactions that do not directly modulate key metabolic pathways [53]. This suggests that pf-*Bj* may contain bioactive compounds with neuroprotective properties that become more effective when administered over extended periods during key stages of neuronal development. This finding is consistent with previous studies showing that peptide-based compounds can modulate antioxidant pathways, such as the activation of the Nrf2/ARE system, thereby promoting a more robust adaptive response to oxidative stress during later developmental stages [1,50,51,52].

The lack of effect from the pf-*Ds* in the late model may indicate structural and functional differences between the peptides in the two fractions, which could influence their ability to activate specific molecular targets [54]. The differential outcomes between pf-*Ds* and pf-*Bj* further suggest that structural and functional differences—such as variations in amino acid sequence or conformational properties—may underlie their distinct abilities to engage molecular targets. These differences are likely to significantly modulate the activation of endogenous neuroprotective pathways, notably the Nrf2/ARE signaling axis, which plays a pivotal role in orchestrating cellular adaptations to oxidative stress [1,50,51,52].

Integrated transcriptomic studies have also demonstrated that the expression of antioxidant and stress-response genes is heavily influenced by developmental stage [55,56], further supporting the notion that the timing of administration is crucial for the neuroprotective efficacy of these compounds. By extending the exposure window, we sought to determine whether these peptides exert their effects primarily through developmental programming mechanisms rather than through immediate antioxidant activity. The use of this second model allowed us to explore a more comprehensive and sustained neuroprotective mechanism, reinforcing the zebrafish as a valuable system for investigating the temporal dynamics and long-term effects of venom-derived peptides in neuroprotection.

While this study provides important initial insights into the biochemical and behavioral safety of peptide fractions derived from *B. jararaca* and *D. siamensis*, some limitations remain to be addressed in future investigations. The current approach prioritized a functional screening strategy to assess neurotoxicity-related endpoints in vivo; however, the precise molecular composition of the fractions has not yet been fully characterized, which limits conclusions regarding structure–activity relationships. In addition, although the selected concentrations and time points were effective for revealing enzymatic and behavioral modulation, further studies are warranted to explore dose–response dynamics, chronic exposure effects, and underlying molecular pathways. Even so, the present findings lay a solid foundation by demonstrating that these venom-derived fractions are safe, biologically active, and promising candidates for further neuroprotective exploration.

## 4. Material and Methods

### 4.1. Reagents and Peptide Fraction of D. siamensis and B. jararaca Venom

All chemicals used in the present study were of analytical reagent grade (purity higher than 95%) and purchased from Gibco BRL (New York, NY, USA), Sigma-Aldrich Corporation (St. Louis, MO, USA), or Synth (Diadema, Brazil). Crude venom of *D. siamensis* and *B. jararaca* was provided by the Laboratory of Herpetology from the Butantan Institute (São Paulo, Brazil), remaining stored at −20 °C until use. The pf-*Ds* and pf-*Bj* were obtained from crude venom (2 g), dissolved, filtered through a Millipore (Billerica, MA, USA) centrifugal filter device with a molecular weight cut-off of 10 kDa, and lyophilized, as previously described [10].

### 4.2. Cell Lines and Maintenance

Neuronal PC12 cells derived from a transplantable rat pheochromocytoma (ATCC^®^ CRL-1721™ from the American Type Culture Collection—ATCC, Manassas, VA, USA) were routinely cultured in DMEM medium (D10; Sigma-Aldrich, St. Louis, MO, USA), supplemented with fetal bovine serum (10% FBS; Gibco, Waltham, MA, USA), penicillin [1% of 10,000 U·mL^−1^ (*v*·*v*^−1^)], streptomycin (10 mg·mL^−1^), and amphotericin B (25 µg·mL^−1^) (Sigma-Aldrich, St. Louis, MO, USA). The cultures were kept at 37 °C in a humidified atmosphere containing 5% CO_2_ (Water Jacketed CO_2_ Incubator, Thermo Scientific, Waltham, MA, USA) and maintained in culture, according to previous reports [8].

#### 4.2.1. Toxicity Studies in Neuronal Cell Lines

The cytotoxic effects of pf-*Ds* were determined by resazurin dye (7-hydroxy-3H-phenoxazin-3-one 10-oxide; Sigma-Aldrich, St. Louis, MO, USA) as an indicator of metabolic activity by resazurin reduction into resorufin [57] and the staining of attached cells with crystal violet dye, according to the literature [58]. Briefly, PC12 cells were seeded into 96-well plates (Nest Biotechnology, Rahway, NJ, USA) at 5 × 10^3^ cells per well and treated with different concentrations (10 to 0.001 µg mL^−1^ of pf-*Ds* diluted in D10) in the presence of resazurin (40 μmol L^−1^; Sigma-Aldrich, St. Louis, MO, USA) in a final volume of 0.10 mL. The plate was incubated at 37 °C, and resorufin fluorescence was assessed by 530 nm excitation and 590 nm emission in a BioTek Synergy microplate reader (BioTek Synergy HT Multi-Mode Microplate Reader, Santa Clara, CA, USA) after 0, 1, 4, and 24 h of treatment. For each concentration and time course studied, there were control and acrylamide (Acr) groups, which represent untreated cells (only one equal volume of the culture medium) and cells treated with Acr 100 mmol L^−1^ diluted in D10, respectively. After that, the medium was aspirated, and the cells were stained with crystal violet staining solution (0.5% (*w*·*v*^−1^)), and the absorbance was measured at 570 nm using a BioTek Epoch microplate spectrophotometer (BioTek Epoch, Santa Clara, CA, USA), according to literature [57,58]. Data were obtained from three independent experiments in sextuplicate and expressed as box-and-whisker plots.

#### 4.2.2. H_2_O_2_-Induced Oxidative Stress Assay

The cellular stress model used in this work was based on H_2_O_2_-induced oxidative stress in neuronal cells, as demonstrated in previous studies [5,8]. Initially, PC12 cells were seeded at 5 × 10^3^ cells per well in a 96-well plate (Nest Biotechnology, Rahway, NJ, USA) and incubated for 24 h. The neuroprotective effects of pf-*Ds* were evaluated in cells that were pretreated at 37 °C for 4 h with pf-*Ds* at a concentration of 1 μg mL^−1^ (a concentration without toxic effects), diluted in D10. Subsequently, the media were replaced with fresh media containing pf-*Ds* and H_2_O_2_ (0.5 mmol L^−1^ in both cell types) and incubated for an additional 20 h (pf-*Ds* + H_2_O_2_ groups). Control cells, which were untreated or those treated with H_2_O_2_ or pf-*Ds*, were also incubated under the same conditions. The neuroprotective effects against H_2_O_2_-induced oxidative stress were then estimated using resazurin and crystal violet dye, as described above [58]. Data were expressed as box-and-whisker plots of cell metabolism or integrity percentage relative to the control.

### 4.3. Zebrafish Maintenance, Husbandry, and Egg Collection

Adult wild-type (WT) strain zebrafish were maintained in the zebrafish bioterium of the Experimental Morphology Laboratory at the Federal University of ABC (UFABC) under the following standard conditions: temperature of 28 °C and light/dark cycle (14/10 h), housed in glass aquariums using distilled water (60 μg mL^−1^, sodium chloride; pH 7.0). The management of zebrafish followed the requirements outlined in the European Directive 2010/63/EU [59] and the National Council for Animal Experimentation Control (CONCEA) [60]. Twice a day, the fish were fed dry food, in addition to artemia nauplii the day before mating. Male and female fish were maintained in equal proportions. To obtain embryos on the designated days, a custom-built apparatus was placed in the aquarium the evening before collection. The fertilized embryos were maintained in E3 medium (5 mmol L^−1^ NaCl, 0.17 mmol L^−1^ KCl, 0.33 mmol L^−1^ CaCl_2_, 0.33 mmol L^−1^ MgSO₄) and kept in an incubator at 28 °C during the experiments.

#### 4.3.1. Monitoring of Zebrafish Development

Zebrafish embryos were harvested at 0 hpf, and treatments (e.g., pf compounds) were administered at 4 hpf. The larvae were exposed to 20, 10, and 1 μg mL^−1^ concentrations of pf-*Ds* (represented by pf-*Ds* 20, pf-*Ds* 10, and pf-*Ds* 1, respectively) or pf-*Bj* (represented by pf-*Bj* 20, pf-*Bj* 10, and pf-*Bj* 1, respectively) diluted in E3 medium and maintained until 120 hpf. The developmental stages were then monitored at multiple time points (8, 24, 48, 72, and 96 hpf). Key evaluation parameters included embryo/larval viability, the onset of morphological abnormalities, and the progression of hatching rate (OECD, 2013) [61]. Individual assessments were performed under a stereomicroscope (LED2500 Leica Microsystems, Wetzlar, Germany). Acrylamide (2 mmol L^−1^) was used as a positive control. For the quantification of enzymatic activity in zebrafish, after 120 h of exposure, the larvae were euthanized by exposure to Tricaine 0.04% (MS-222, Sigma-Aldrich) until total absence of movement, homogenized with chilled phosphate buffer (0.1 mmol mL^−1^), and centrifuged for 15 min at 5000 rpm. The supernatant was collected, and the protein content of the sample was determined using the Bradford method [62]. Subsequently, parameters assessing oxidative stress and acetylcholinesterase activity were analyzed. Superoxide dismutase (SOD) activity was determined based on its ability to inhibit a superoxide radical-dependent reaction [63,64]. Catalase (CAT) activity was assessed by monitoring the rate of hydrogen peroxide (H_2_O_2_) decomposition [65]. Finally, acetylcholinesterase (AChE) activity was quantified based on the Ellman method [66].

#### 4.3.2. Neuroprotection Assay Against Oxidative Stress

Initially, zebrafish embryos were collected at 0 hpf and maintained under standard conditions until 96 hpf, when treatments with either pf-*Ds* or pf-*Bj* were administered at a predetermined concentration (10 μg mL^−1^) in E3 medium. After 4 h (at 100 hpf), H_2_O_2_ (0.2 mmol L^−1^) was added alongside the peptide fractions to induce oxidative stress. Final assessments, including metabolic and locomotor activity analyses, were performed at 120 hpf. This experimental approach was referred to as the “4–20 h model”. The metabolic activity of zebrafish larvae was estimated by the reduction of resazurin to resorufin (7-hydroxy-3H-phenoxazin-3-one 10-oxide; Sigma-Aldrich, St. Louis, MO, USA) [57]. Solutions containing the larvae from the previously mentioned experimental groups were supplemented with 80 μmol L^−1^ of resazurin. After 24 h (at 5 dpf), the fluorescence of resorufin was evaluated by excitation at 530 nm and emission at 590 nm using a microplate reader (BioTek, Winooski, VT, USA) and expressed as a percentage of the control. To evaluate the effects of prolonged exposure, we followed a modified version based on the previous protocol [16]. This protocol involved exposing zebrafish embryos at 4 hpf to the predetermined neuroprotection test concentration of pf-*Ds* and pf-*Bj* (10 μg mL^−1^) in E3 medium. The larvae were then incubated for 96 h, reaching 100 hpf, at which point, the peptide fraction solutions were entirely replaced with either an H_2_O_2_ (0.2 mmol L^−1^) solution diluted in E3 medium or only E3 medium for the control groups for an additional 24 h. This experimental approach was referred to as the “96–120 h model”. Following this period, the larvae were recorded for behavioral analysis and subsequently processed for metabolic assessment, as well as the quantification of superoxide dismutase (SOD), catalase (CAT), and acetylcholinesterase (AChE), as previously described.

#### 4.3.3. Behavior Analysis

Locomotor activity was investigated by analyzing the swimming behavior of 120 hpf zebrafish larvae [17]. Treated or non-treated groups (*n* = 12) were transferred to 96-well plates, with one larva per well in 100 μL of E3 medium, and the videos were captured using a custom-built recording apparatus. After a minimum 15 min acclimatization period, the larvae were analyzed for a total of 160 s, and the locomotor activity was quantified and analyzed using ImageJ2 and Fiji software [67,68]. The total distance results were obtained by summing the distances moved, and the total average speed was obtained by dividing the distance by the analysis time.

### 4.4. Statistical Analyses

Data were analyzed using one-way analysis of variance (ANOVA) for between-group comparisons, followed by Tukey’s post hoc test for multiple comparisons or Dunnett’s post hoc test to compare each of some treatments with a single control or Student’s *t*-test. Values of *p* < 0.05 were statistically significant. The analyses were performed using GraphPad Prism 8.0 software (GraphPad Software, Inc., La Jolla, CA, USA).

## 5. Conclusions

Overall, our findings underscore that the neuroprotective efficacy of peptide fractions is dependent on both the duration of exposure and the stage of neuronal maturation. The increased efficacy of pf-*Bj* in the late-stage model highlights the importance of administering these compounds at a developmental time point when the endogenous antioxidant systems are fully operational, an insight that is critical for translating these findings into future biomedical applications. While this study prioritized functional outcomes, future research should focus on the comprehensive molecular characterization of these fractions and the identification of their specific molecular targets, which will be essential for the rational development of novel neuroactive agents inspired by snake venom-derived peptides.

## Data Availability

All data generated or analyzed during this study are included in this published article.

## References

[1] Nguyen T., Nioi P., Pickett C.B. (2009). The Nrf2-Antioxidant Response Element Signaling Pathway and Its Activation by Oxidative Stress. J. Biol. Chem..

[2] Abbate F., Maugeri A., Laurà R., Levanti M., Navarra M., Cirmi S., Germanà A. (2021). Zebrafish as a Useful Model to Study Oxidative Stress-Linked Disorders: Focus on Flavonoids. Antioxidants.

[3] Hussain S., Slikker W., Ali S.F. (1995). Age-Related Changes in Antioxidant Enzymes, Superoxide Dismutase, Catalase, Glutathione Peroxidase and Glutathione in Different Regions of Mouse Brain. Int. J. Dev. Neurosci..

[4] Wang P., Zhang Y., Hu J., Tan B.K. (2024). Bioactive Peptides from Marine Organisms. Protein Pept. Lett..

[5] Alberto-Silva C., da Silva B.R., da Silva J.C.A., Cunha e Silva F.A., da Kodama R.T., da Silva W.D., Costa M.S., Portaro F.C.V. (2024). Small Structural Differences in Proline-Rich Decapeptides Have Specific Effects on Oxidative Stress-Induced Neurotoxicity and L-Arginine Generation by Arginosuccinate Synthase. Pharmaceuticals.

[6] Lima C., Disner G.R., Falcão M.A.P., Seni-Silva A.C., Maleski A.L.A., Souza M.M., Tonello M.C.R., Lopes-Ferreira M. (2021). The Natterin Proteins Diversity: A Review on Phylogeny, Structure, and Immune Function. Toxins.

[7] Boix N., Teixido E., Pique E., Llobet J.M., Gomez-Catalan J. (2020). Modulation and Protection Effects of Antioxidant Compounds against Oxidant Induced Developmental Toxicity in Zebrafish. Antioxidants.

[8] Alberto-Silva C., Pantaleão H.Q., da Silva B.R., da Silva J.C.A., Echeverry M.B. (2024). Activation of M1 Muscarinic Acetylcholine Receptors by Proline-Rich Oligopeptide 7a (<EDGPIPP) from *Bothrops jararaca* Snake Venom Rescues Oxidative Stress-Induced Neurotoxicity in PC12 Cells. J. Venom. Anim. Toxins Incl. Trop. Dis..

[9] Pantaleão H.Q., da Araujo Silva J.C., da Rufino Silva B., Echeverry M.B., Alberto-Silva C. (2023). Peptide Fraction from *B. jararaca* Snake Venom Protects against Oxidative Stress-Induced Changes in Neuronal PC12 Cell but Not in Astro-cyte-like C6 Cell. Toxicon.

[10] Querobino S.M., Carrettiero D.C., Costa M.S., Alberto-Silva C. (2017). Neuroprotective Property of Low Molecular Weight Fraction from *B. jararaca* Snake Venom in H_2_O_2_-Induced Cytotoxicity in Cultured Hippocampal Cells. Toxicon.

[11] Kimmel C.B., Ballard W.W., Kimmel S.R., Ullmann B., Schilling T.F. (1995). Stages of Embryonic Development of the Zebrafish. Dev. Dyn..

[12] Kalueff A.V., Stewart A.M., Gerlai R. (2014). Zebrafish as an Emerging Model for Studying Complex Brain Disorders. Trends Pharmacol. Sci..

[13] Irons T.D., MacPhail R.C., Hunter D.L., Padilla S. (2010). Acute Neuroactive Drug Exposures Alter Locomotor Activity in Larval Zebrafish. Neurotoxicol. Teratol..

[14] Hill A.J., Teraoka H., Heideman W., Peterson R.E. (2005). Zebrafish as a Model Vertebrate for Investigating Chemical Tox-icity. Toxicol. Sci..

[15] Maleski A.L.A., Rosa J.G.S., Bernardo J.T.G., Astray R.M., Walker C.I.B., Lopes-Ferreira M., Lima C. (2022). Recapitulation of Retinal Damage in Zebrafish Larvae Infected with Zika Virus. Cells.

[16] Issac P.K., Guru A., Velayutham M., Pachaiappan R., Arasu M.V., Al-Dhabi N.A., Choi K.C., Harikrishnan R., Arockiaraj J. (2021). Oxidative Stress Induced Antioxidant and Neurotoxicity Demonstrated in Vivo Zebrafish Embryo or Larval Model and Their Normalization Due to Morin Showing Therapeutic Implications. Life Sci..

[17] Rosa J.G.S., Lima C., Lopes-Ferreira M. (2022). Zebrafish Larvae Behavior Models as a Tool for Drug Screenings and Pre-Clinical Trials: A Review. Int. J. Mol. Sci..

[18] Alberto-Silva C., da Silva B.R. (2024). Molecular and Cellular Mechanisms of Neuroprotection by Oligopeptides from Snake Venoms. Biocell.

[19] Alberto-Silva C., Portaro F.C.V. (2024). Neuroprotection Mediated by Snake Venom. Natural Molecules in Neuroprotection and Neurotoxicity.

[20] Silva B.R., Mendes L.C., Echeverry M.B., Juliano M.A., Beraldo-Neto E., Alberto-Silva C. (2025). Peptide Fraction from Naja Mandalayensis Snake Venom Showed Neuroprotection Against Oxidative Stress in Hippocampal MHippoE-18 Cells but Not in Neuronal PC12 Cells. Antioxidants.

[21] Cervelli M., Averna M., Vergani L., Pedrazzi M., Amato S., Fiorucci C., Rossi M.N., Maura G., Mariottini P., Cervetto C. (2022). The Involvement of Polyamines Catabolism in the Crosstalk between Neurons and Astrocytes in Neurodegeneration. Biomedicines.

[22] Gogoi M., Datey A., Wilson K.T., Chakravortty D. (2016). Dual Role of Arginine Metabolism in Establishing Pathogenesis. Curr. Opin. Microbiol..

[23] Jamwal S., Kumar P. (2016). Spermidine Ameliorates 3-Nitropropionic Acid (3-NP)-Induced Striatal Toxicity: Possible Role of Oxidative Stress, Neuroinflammation, and Neurotransmitters. Physiol. Behav..

[24] Kotagale N.R., Taksande B.G., Inamdar N.N. (2019). Neuroprotective Offerings by Agmatine. Neurotoxicology.

[25] Madeo F., Eisenberg T., Pietrocola F., Kroemer G. (2018). Spermidine in Health and Disease. Science.

[26] Haas J., Storch-Hagenlocher B., Biessmann A., Wildemann B. (2002). Inducible nitric oxide synthase and argininosuccinate synthetase: Co-induction in brain tissue of patients with Alzheimer’s dementia and following stimulation with beta-amyloid 1–42 in vitro. Neurosci. Lett..

[27] Casewell N.R., Jackson T.N.W., Laustsen A.H., Sunagar K. (2020). Causes and Consequences of Snake Venom Variation. Trends Pharmacol. Sci..

[28] Amorim A.M.B., Piochi L.F., Gaspar A.T., Preto A.J., Rosário-Ferreira N., Moreira I.S. (2024). Advancing Drug Safety in Drug Development: Bridging Computational Predictions for Enhanced Toxicity Prediction. Chem. Res. Toxicol..

[29] Barros T.P., Alderton W.K., Reynolds H.M., Roach A.G., Berghmans S. (2008). Zebrafish: An Emerging Technology for in Vivo Pharmacological Assessment to Identify Potential Safety Liabilities in Early Drug Discovery. Br. J. Pharmacol..

[30] McGrath P., Li C.Q. (2008). Zebrafish: A Predictive Model for Assessing Drug-Induced Toxicity. Drug Discov. Today.

[31] Naik R.A., Mir M.N., Malik I.A., Bhardwaj R., Alshabrmi F.M., Mahmoud M.A., Alhomrani M., Alamri A.S., Alsanie W.F., Hjazi A. (2025). The Potential Mechanism and the Role of Antioxidants in Mitigating Oxidative Stress in Alzheimer’s Disease. Front. Biosci. (Landmark Ed.).

[32] Pizzino G., Irrera N., Cucinotta M., Pallio G., Mannino F., Arcoraci V., Squadrito F., Altavilla D., Bitto A. (2017). Oxidative Stress: Harms and Benefits for Human Health. Oxid. Med. Cell. Longev..

[33] Bertrand C., Chatonnet A., Takke C., Yan Y.L., Postlethwait J., Toutant J.P., Cousin X. (2001). Zebrafish Acetylcholinesterase Is Encoded by a Single Gene Localized on Linkage Group 7. Gene Structure and Polymorphism; Molecular Forms and Expression Pattern during Development. J. Biol. Chem..

[34] Richetti S.K., Rosemberg D.B., Ventura-Lima J., Monserrat J.M., Bogo M.R., Bonan C.D. (2011). Acetylcholinesterase Activity and Antioxidant Capacity of Zebrafish Brain Is Altered by Heavy Metal Exposure. Neurotoxicology.

[35] Sant’Anna M.C.B., de Soares V.M., Seibt K.J., Ghisleni G., Rico E.P., Rosemberg D.B., de Oliveira J.R., Schrö-der N., Bonan C.D., Bogo M.R. (2011). Iron Exposure Modifies Acetylcholinesterase Activity in Zebrafish (*Danio rerio*) Tissues: Distinct Susceptibility of Tissues to Iron Overload. Fish Physiol. Biochem..

[36] Radi R. (2018). Oxygen Radicals, Nitric Oxide, and Peroxynitrite: Redox Pathways in Molecular Medicine. Proc. Natl. Acad. Sci. USA.

[37] Valko M., Leibfritz D., Moncol J., Cronin M.T.D., Mazur M., Telser J. (2007). Free Radicals and Antioxidants in Normal Physiological Functions and Human Disease. Int. J. Biochem. Cell Biol..

[38] Barber S.C., Shaw P.J. (2010). Oxidative Stress in ALS: Key Role in Motor Neuron Injury and Therapeutic Target. Free. Radic. Biol. Med..

[39] Dias V., Junn E., Mouradian M.M. (2013). The Role of Oxidative Stress in Parkinson’s Disease. J. Park. Dis..

[40] Martínez-Reyes I., Chandel N.S. (2020). Mitochondrial TCA Cycle Metabolites Control Physiology and Disease. Nat. Commun..

[41] Slotkin T.A. (2004). Cholinergic Systems in Brain Development and Disruption by Neurotoxicants: Nicotine, Environmental Tobacco Smoke, Organophosphates. Toxicol. Appl. Pharmacol..

[42] Nordberg A., Svensson A.-L. (1998). Cholinesterase Inhibitors in the Treatment of Alzheimer’s Disease. Drug Saf..

[43] Khademi N., Mehrnia N., Roshan M.E. (2024). Bioactive Peptides: A Complementary Approach for Cancer Therapy. Asian Pac. J. Cancer Care.

[44] Zhang D.L., Hu C.X., Li D.H., Liu Y.D. (2013). Lipid Peroxidation and Antioxidant Responses in Zebrafish Brain Induced by Aphanizomenon Flos-Aquae DC-1 Aphantoxins. Aquat. Toxicol..

[45] Lee H.J., Hou Y., Maeng J.H., Shah N.M., Chen Y., Lawson H.A., Yang H., Yue F., Wang T. (2022). Epigenomic Analysis Reveals Prevalent Contribution of Transposable Elements to Cis -Regulatory Elements, Tissue-Specific Expression, and Alternative Promoters in Zebrafish. Genome Res..

[46] Dyker A.G., Lees K.R. (1998). Duration of Neuroprotective Treatment for Ischemic Stroke. Stroke.

[47] Lee B., Butcher G.Q., Hoyt K.R., Impey S., Obrietan K. (2005). Activity-Dependent Neuroprotection and CAMP Response Element-Binding Protein (CREB): Kinase Coupling, Stimulus Intensity, and Temporal Regulation of CREB Phosphorylation at Serine 133. J. Neurosci..

[48] Martins N.M., Santos N.A.G., Sartim M.A., Cintra A.C.O., Sampaio S.V., Santos A.C. (2015). A Tripeptide Isolated from Bothrops Atrox Venom Has Neuroprotective and Neurotrophic Effects on a Cellular Model of Parkinson’s Disease. Chem. Biol. Interact..

[49] Salem F.E., Yehia H.M., Korany S.M., Alarjani K.M., Al-Masoud A.H., Elkhadragy M.F. (2022). Neurotherapeutic Effects of Prodigiosin Conjugated with Silver-Nanoparticles in Rats Exposed to Cadmium Chloride-Induced Neurotoxicity. Food Sci. Technol. (Campinas).

[50] Hahn M.E., Timme-Laragy A.R., Karchner S.I., Stegeman J.J. (2015). Nrf2 and Nrf2-Related Proteins in Development and Developmental Toxicity: Insights from Studies in Zebrafish (*Danio rerio*). Free Radic. Biol. Med..

[51] Marques E.S., Severance E.G., Arsenault P., Zahn S.M., Timme-Laragy A.R. (2024). Activation of Nrf2 at Critical Windows of Development Alters Tissue-Specific Protein S-Glutathionylation in the Zebrafish (*Danio rerio*) Embryo. Antioxidants.

[52] Staurengo-Ferrari L., Badaro-Garcia S., Hohmann M.S.N., Manchope M.F., Zaninelli T.H., Casagrande R., Verri W.A. (2019). Contribution of Nrf2 Modulation to the Mechanism of Action of Analgesic and Anti-Inflammatory Drugs in Pre-Clinical and Clinical Stages. Front. Pharmacol..

[53] Sun D., Gao W., Hu H., Zhou S. (2022). Why 90% of Clinical Drug Development Fails and How to Improve It?. Acta Pharm. Sin. B.

[54] Ciulla M.G., Gelain F. (2023). Structure-Activity Relationships of Antibacterial Peptides. Microb. Biotechnol..

[55] Wu K.C., Cui J.Y., Liu J., Lu H., Zhong X., Klaassen C.D. (2019). RNA-Seq Provides New Insights on the Relative MRNA Abundance of Antioxidant Components during Mouse Liver Development. Free. Radic. Biol. Med..

[56] Mills M.G., Gallagher E.P. (2017). A Targeted Gene Expression Platform Allows for Rapid Analysis of Chemical-Induced Antioxidant MRNA Expression in Zebrafish Larvae. PLoS ONE.

[57] Borra R.C., Lotufo M.A., Gagioti S.M., de Barros F.M., Andrade P.M. (2009). A Simple Method to Measure Cell Viability in Proliferation and Cytotoxicity Assays. Braz. Oral Res..

[58] Feoktistova M., Geserick P., Leverkus M. (2016). Crystal Violet Assay for Determining Viability of Cultured Cells. Cold Spring Harb. Protoc..

[59] European Parliament and Council of the European Union (2010). Directive 2010/63/EU of the European Parliament and of the Council of 22 September 2010 on the Protection of Animals Used for Scientific Purposes.

[60] Conselho Nacional de Controle de Experimentação Animal (CONCEA) (2023). Capítulo 5—Lambari, tilápia e zebrafish. Guia Brasileiro de Produção, Manutenção ou Utilização de Animais em Atividades de Ensino ou Pesquisa Científica.

[61] OECD (2013). Test No. 236: Fish Embryo Acute Toxicity (FET) Test.

[62] Bradford M.M. (1976). A Rapid and Sensitive Method for the Quantitation of Microgram Quantities of Protein Utilizing the Principle of Protein-Dye Binding. Anal. Biochem..

[63] Silva R.F.O., Pinho B.R., Santos M.M., Oliveira J.M.A. (2022). Disruptions of Circadian Rhythms, Sleep, and Stress Responses in Zebrafish: New Infrared-Based Activity Monitoring Assays for Toxicity Assessment. Chemosphere.

[64] Marklund S., Marklund G. (1974). Involvement of the Superoxide Anion Radical in the Autoxidation of Pyrogallol and a Convenient Assay for Superoxide Dismutase. Eur. J. Biochem..

[65] Hadwan M.H. (2018). Simple Spectrophotometric Assay for Measuring Catalase Activity in Biological Tissues. BMC Biochem..

[66] Padilla S., Lassiter T.L., Hunter D.L., Harry G.J., Tilson H.A. (1999). Biochemical Measurement of Cholinesterase Activity. Neurodegeneration Methods and Protocols.

[67] Rueden C.T., Schindelin J., Hiner M.C., DeZonia B.E., Walter A.E., Arena E.T., Eliceiri K.W. (2017). ImageJ2: ImageJ for the next Generation of Scientific Image Data. BMC Bioinform..

[68] Schindelin J., Arganda-Carreras I., Frise E., Kaynig V., Longair M., Pietzsch T., Preibisch S., Rueden C., Saalfeld S., Schmid B. (2012). Fiji: An Open-Source Platform for Biological-Image Analysis. Nat. Methods.

